# Cardiorespiratory fitness is associated with hippocampal resting state connectivity in women newly diagnosed with breast cancer

**DOI:** 10.3389/fcogn.2023.1211525

**Published:** 2023-08-03

**Authors:** Alina Lesnovskaya, Hayley S. Ripperger, Shannon D. Donofry, Jermon A. Drake, Lu Wan, Alexa Poniatowski, Patrick T. Donahue, Mary E. Crisafio, Alysha D. Gilmore, Emily A. Richards, George Grove, Amanda L. Gentry, Susan M. Sereika, Catherine M. Bender, Kirk I. Erickson

**Affiliations:** 1 Department of Psychology, University of Pittsburgh, Pittsburgh, PA, United States,; 2 Center for the Neural Basis of Cognition, University of Pittsburgh and Carnegie Mellon University, Pittsburgh, PA, United States,; 3 Institute for Graduate Clinical Psychology, Widener University, Chester, PA, United States,; 4 Department of Mental Health, Johns Hopkins Bloomberg School of Public Health, Baltimore, MD, United States,; 5 Department of Health and Exercise Science, Colorado State University, Fort Collins, CO, United States,; 6 School of Nursing, University of Pittsburgh, Pittsburgh, PA, United States,; 7 Graduate School of Public Health, University of Pittsburgh, Pittsburgh, PA, United States,; 8 Clinical and Translational Science Institute, University of Pittsburgh, Pittsburgh, PA, United States,; 9 Department of Neuroscience, AdventHealth Research Institute, Orlando, FL, United States

**Keywords:** hippocampus, breast cancer, cognition, fMRI, resting state functional connectivity, cardiorespiratory fitness

## Abstract

****Background**:**

Breast cancer and its treatment are associated with aberrant patterns of resting state functional connectivity (rsFC) between the hippocampus and several areas of the brain, which may account for poorer cognitive outcomes in patients. Higher cardiorespiratory fitness (CRF) has been associated with enhanced rsFC and cognitive performance; however, these associations have not been well studied in breast cancer. We examined the relationship between CRF, rsFC of the hippocampus, and cognitive performance among women newly diagnosed with breast cancer.

****Methods**:**

Thirty-four postmenopausal women newly diagnosed with Stage 0-IIIa breast cancer (*M*_age_ = 63.59 ± 5.73) were enrolled in a 6-month randomized controlled trial of aerobic exercise vs. usual care. During baseline assessments, participants completed functional brain imaging, a submaximal CRF test, and cognitive testing. Whole-brain, seed-based analyses were used to examine the relationship between CRF and hippocampal rsFC, with age, years of education, and framewise displacement included as covariates. Cognition was measured with a battery of validated neurocognitive measures, reduced to seven composite factors.

****Results**:**

Higher CRF was positively associated with greater rsFC of the hippocampus to a cluster within the dorsomedial and dorsolateral frontal cortex (*z*-max = 4.37, *p* = 0.003, cluster extent = 1,020 voxels). Connectivity within cluster peaks was not significantly related to cognitive factors (all *p*s > 0.05).

****Discussion**:**

CRF was positively associated with hippocampal rsFC to frontal cortex structures, comprising a network of regions commonly suppressed in breast cancer. Future longitudinal research is needed to explore whether baseline rsFC predicts long-term cognitive resilience in breast cancer.

## Introduction

Breast cancer is associated with altered brain function and poorer cognitive performance, particularly in aspects of cognition mediated by frontal and medial temporal brain activity, including memory and executive function (Ahles et al., 2008; Quesnel et al., 2009; Hermelink et al., 2015; Ono et al., 2015). Studies using functional magnetic resonance imaging (fMRI) to investigate the neural correlates of cognitive decline in breast cancer have reported reduced brain activation, both at rest and during cognitive task performance, in regions including the hippocampus and prefrontal cortex (PFC), with further decline following adjuvant therapy (López Zunini et al., 2013; Peukert et al., 2020). Moreover, up to 75% of breast cancer survivors are estimated to experience cognitive decline, the degree of which is associated with lower self-reported quality of life (Ahles et al., 2012; Henderson et al., 2019) and may predict long-term risk for Alzheimer’s disease (Kesler et al., 2017). Unfortunately, there is no current treatment for neurocognitive decline in breast cancer, indicating a need for research investigating how modifiable factors may mitigate cancer-related dysregulation in brain function.

In particular, the hippocampus has been shown to be vulnerable to both structural and functional decline in the context of cancer treatment (de Ruiter et al., 2011). For instance, several studies focusing on structural brain health have reported that breast cancer survivors have smaller hippocampal volumes and associated reduced memory function as compared to women without cancer (Bergouignan et al., 2011; Kesler et al., 2013; McDonald and Saykin, 2013). Furthermore, several studies have reported that patients who underwent treatment for breast cancer demonstrated altered patterns of resting state functional connectivity (rsFC) between the hippocampus and prefrontal cortex (Chen et al., 2017; Apple et al., 2018; Feng et al., 2020). For example, Chen et al. (2017) showed that women with breast cancer who were treated with tamoxifen had decreased connectivity between the dorsolateral prefrontal cortex (DLPFC) and the hippocampus, as well as significantly poorer working memory and executive function performance, compared with healthy controls. Of note, rsFC is a functional magnetic resonance imaging (fMRI) approach which measures temporal correlations in the blood oxygenation (thought to represent underlying neural signal) of spatially distributed brain areas during periods of rest, with the assumption that brain regions that co-activate at rest form functional networks that support various cognitive processes. Thus, such aberrant patterns of hippocampal rsFC in breast cancer may contribute to reduced cognitive performance and the trajectory of long-term brain outcomes (Kardan et al., 2019). This is particularly relevant in the context of late adulthood, as the majority of breast cancer cases are diagnosed post-menopause (Heer et al., 2020).

Nevertheless, there is a lack of research examining modifiable bio-behavioral variables that may be leveraged to prevent or mitigate dysregulation in hippocampal rsFC and related cognitive decline in breast cancer. One such factor is cardiorespiratory fitness, a physiological measure which has had promising brain-protective associations across numerous populations, both clinically and in the context of healthy aging (Aghjayan et al., 2021). Increasing cardiorespiratory fitness via engagement in regular aerobic exercise is thought to benefit the structural and functional health of the brain through several possible avenues, including reducing inflammation and increasing neuronal growth factor expression (Molteni et al., 2004; Ghiani et al., 2007; Morel et al., 2017). Furthermore, rsFC has been identified as a key source of variance in the relationship between cardiorespiratory fitness and cognition in healthy adults (Voss et al., 2010; Stillman et al., 2018, 2019). With respect to breast cancer, engagement in physical activity and higher levels of cardiorespiratory fitness have been associated with better cognitive performance (Bender et al., 2020) and greater hippocampal volume (Chaddock-Heyman et al., 2015), but the neural underpinnings of this association are not well understood. Moreover, the relationship between cardiorespiratory fitness and rsFC of the hippocampus has not yet been examined in women with breast cancer.

Thus, the purpose of the current investigation was to examine the relationship between cardiorespiratory fitness and hippocampal rsFC, as well as associations with cognitive performance, in a sample of postmenopausal women with early-stage breast cancer who have recently begun or are about to begin adjuvant therapy. Based on findings from investigations of cardiorespiratory fitness and hippocampal rsFC in healthy adults (Stillman et al., 2018; Kronman et al., 2020), we predicted that higher cardiorespiratory fitness would be associated with patterns of greater rsFC between the hippocampus and areas within the prefrontal cortex, and that these associations would be associated with better memory and executive function performance.

## Materials and methods

### Participants

Participants were postmenopausal women newly diagnosed with Stage 0-IIIa breast cancer who were enrolled in a 6-month randomized controlled trial of aerobic exercise and neurocognitive function (ClinicalTrials.gov NCT02793921). Eligible participants were required to be diagnosed with hormone receptor-positive breast cancer as confirmed by their medical oncologist; “newly diagnosed” was defined as being within 2 years of completion of primary treatment. The goal was to assess participants after surgery but prior to beginning adjuvant therapy. However, some participants were assessed after starting aromatase inhibitor (AI) therapy (Table 2). Exclusion criteria included being premenopausal, prior treatment with cancer chemotherapy, central nervous system radiation, or intrathecal therapy, clinical evidence of distant metastases, breast cancer surgery complications (e.g., wound dehiscence), ineligibility for AI therapy, recent falls or use of an assisted walking device, comorbid medical conditions that would preclude engagement in aerobic exercise (e.g., coronary artery or pulmonary disease), current use of hormone replacement therapy, history of neurologic conditions (e.g., stroke), hospitalization for a psychiatric illness in the previous 2 years, substance use or eating disorder history, and age above 80 years. MRI specific exclusion criteria included claustrophobia and presence of irremovable metal implants. The present analysis utilized data acquired during the baseline assessments. The participants for this analysis were among a subsample of women who were eligible for and consented to undergo fMRI scanning (*N* = 34). Informed consent was obtained in accordance with the guidelines of the University of Pittsburgh and Carnegie Mellon University Institutional Review Boards. Baseline neuroimaging assessments were conducted between November 2016 and December 2019.

### Assessments

#### Cardiorespiratory fitness

Cardiorespiratory fitness was measured using a sub-maximal graded exercise test to assess aerobic capacity using a modified Balke protocol. This required walking on a motorized treadmill at a constant speed between 2.0 and 4.0 miles per hour (mph) as the grade increased by 1% each minute. Test speed determined by the ability of the participant in consultation with the exercise physiologist at increments of 0.5 mph within the 2.0–4.0 mph range. Prior to the test, height in inches and body mass in pounds were recorded and the participant was fitted with a facemask to collect expired air. The test was terminated if any of the following criteria were met: (1) the participant reached 85% of the age-predicted maximal heart rate (220—age); (2) the participant endorsed a Borg rating (Borg, 1982) of perceived exertion of 15 or greater in participants taking a beta blocker medication; (3) the participant reached volitional exhaustion; or (4) the monitoring medical team expressed safety concerns about continuation of the test. Oxygen consumption was analyzed using a ParvoMedics metabolic cart. VO_2_submax was defined as the oxygen consumption relative to body weight in kilograms (ml/kg/min) achieved at 85% of the age-predicted maximal heart rate.

#### Cognitive assessments

Neurocognitive function was measured with a battery of validated neurocognitive measures in the larger sample (*N* = 121) from which this MRI sub-study is derived. Measures were selected due to their demonstrated sensitivity to change in multiple neurocognitive domains in women with breast cancer (Bender et al., 2015). Due to the high number of scores produced from this battery, an empirically-based exploratory factor analysis with orthogonal rotation was conducted to reduce the number of outcome variables. This procedure yielded seven factors, outlined in Table 1. Mean z-scores for the seven factors were computed as the average of the participant’s standardized scores from individual tests in the battery of neurocognitive measures and relative to normative data from women without breast cancer, matched on age and education. Higher positive z-scores indicate better performance relative to the control group at baseline.

#### MRI data acquisition and pre-processing

The methods used for the MRI data acquisition and pre-processing for this sample have been previously described (Donofry et al., 2022). MRI sequences were collected using a Siemens Verio 3T scanner (Siemens Medical Solutions USA, Inc., Malvern, PA) with a 32-channel head coil. For resting state imaging, 210 *T*2*-weighted volumes were obtained for each participant using an EPI pulse sequence with blood oxygenation level-dependent (BOLD) contrast (time repetition = 1,540 ms, echo time = 25 ms, flip angle = 90^◦^). Thirty slices were collected at 3.5 mm thickness in the axial plane in the ventral to dorsal direction. High resolution *T*1-weighted anatomical volumes were also collected in the sagittal plane using a magnetization-prepared rapid gradient-echo (MPRAGE) sequence for each participant (256 slices, voxel dimensions 1 × 0.976 × 0.976 mm). After reconstruction, data were preprocessed using FEAT version 6.0, part of FSL (FMRIB’s Software Library; http://www.fmrib.ox.ac.uk/fsl/). The MCFLIRT (Jenkinson et al., 2002) tool was used to adjust for between-volume motion, with the middle image designated as the reference. A threshold of 1.7 mm displacement in any direction was used to identify motion spikes or excessive movement. A bandpass temporal filter between 0.001 and 0.01 Hz was applied to resting state volumes to remove high frequency noise and low frequency noise related to scanner drift. Images were spatially smoothed with a 6-mm full-width half-maximum 3-dimensional Gaussian kernel. Non-brain matter was removed using the robust brain extraction technique (BET) (Smith, 2002). Mean functional images were registered to *T*1-weighted images and MNI space (Montreal Neurological Institute—International Consortium for Brain Mapping) images applying 7- and 12-parameter affine transformations respectively using FMRIB’s linear image registration tool (FLIRT) (Jenkinson and Smith, 2001; Jenkinson et al., 2002). No errors were identified during image registration.

#### Resting state functional connectivity analyses

A seed-based approach was used to assess whole-brain resting state functional connectivity, with the hippocampus designated as a seed and structurally defined using the Harvard-Oxford Subcortical Atlas. Using the FSL FIRST tool (Patenaude et al., 2011), automated segmentation of subcortical structures was performed to define the seed mask for the hippocampus. Masks for the left and right hippocampus were merged to create a mask representing the total structure of the hippocampal seed. The hippocampal seed mask was then converted to native space for each individual, after which the BOLD signal time series for the seed was extracted from each individual’s preprocessed data. The mean time course of the seed was entered into a multiple linear regression model to estimate BOLD signal covariation between the seed and all voxels across the brain. A contrast parameter was created to examine which voxels positively and negatively covaried with the seed region time series. Individual-level general linear models (GLMs) included the global signal, signal from white matter and cerebrospinal fluid, as well as standard and extended estimates of motion displacement (standard motion parameters with their derivatives and the square of their derivatives) as regressors of no interest. Beta values derived from these individual-level analyses were then forwarded to higher level GLMs to examine whether cardiorespiratory fitness was associated with variation in seed-to-voxel correlations at rest. Age, education, and mean framewise displacement values were included as covariates in group-level models. A voxelwise threshold of *p* < 0.01, and cluster-defining family-wise error threshold of *p* < 0.05 were applied to all statistical parametric maps generated for each contrast.

#### Relationship between resting connectivity, cardiorespiratory fitness, and cognitive performance

To assess the relationship between estimates of hippocampal functional connectivity and cardiorespiratory fitness, regions of interest (ROIs) were first identified based on local maxima within clusters exhibiting significant cardiorespiratory fitness-dependent signal covariation with the hippocampus seed region, which were then selected for further analysis. For each participant, covariation between the time series of each identified ROI and the time series of the seed region were estimated using pairwise correlation coeffcients, which were then normalized using a Fisher *r*-to-*z* transformation. The FSL featquery function was used to extract the mean parameter estimates within a 10 mm sphere around local maxima coordinates. Parameter estimates were then exported to a database that included clinical and demographic data. Descriptive analyses were conducted to examine sample characteristics. Multiple linear regression analyses were then performed to examine whether (1) cardiorespiratory fitness was associated with any composite cognitive factors and (2) seed-to-ROI resting state connectivity values were associated with composite cognitive factors. Age and years of education were included as covariates in all models. Analyses were conducted in R Studio version 1.2.1335 and scatterplots were created using the package “ggplot2”.

## Results

### Sample characteristics

Demographic characteristics of the neuroimaging sample (N = 34) are summarized in Table 2. As previously noted, the participants for this analysis were among a subsample of women enrolled in a randomized controlled trial of exercise vs. usual care, who were eligible for and consented to undergo fMRI scanning. Women who completed fMRI assessments did not differ significantly (*p* < 0.05) from the larger overall sample enrolled to date on any clinical (i.e., cardiorespiratory fitness, mood, anxiety, or cancer-related) or demographic indicator. The age distribution of the neuroimaging sample ranged from 51 to 76. Participants were predominantly White (82%) and non-Hispanic (97%). Mean BMI for the sample was in the obese range (*M* = 31.83, *SD* = 6.62), with 85% (*N* = 29) of women having a BMI of 25 or greater. There were no significant associations between VO_2_submax and age (*r* =−0.16, *p* = 0.38) or years of education (*r* = −0.22, *p* = 0.21), and VO_2_submax did not differ by race *t*_(10.16)_ = −0.17, *p* = 0.87.

It was not always possible to have participants complete their MRI or cognitive testing assessment prior to starting AI therapy or starting the intervention. In total, 91% (*N* = 31) of the participants underwent baseline MRI before or within 3 weeks of starting AI therapy. The three participants who underwent baseline MRI at least 3 weeks after starting AI therapy completed imaging 45, 219, and 525 days following the start of treatment, respectively (Table 2). These same three participants also completed the cognitive testing assessment after starting AI therapy (22, 195, and 511 days following the start of treatment). These three participants were excluded from sensitivity analyses, described below. Finally, there were 11 participants who had begun the exercise intervention prior to undergoing their baseline MRI. The maximum number of days between starting the exercise intervention and MRI was 15 days. Importantly, the first 2 weeks of the exercise intervention was an orientation period that entailed exercising for only 10–15 min, three times per week. We did not expect this amount of exercise to influence rsFC. Nonetheless, we conducted *t*-tests comparing the rsFC values of the 11 participants who had started exercising to the 23 participants who had not, and there were no significant differences between the two groups for any of the ROIs.

### Cardiorespiratory fitness and hippocampal rsFC

**FIGURE 1:**
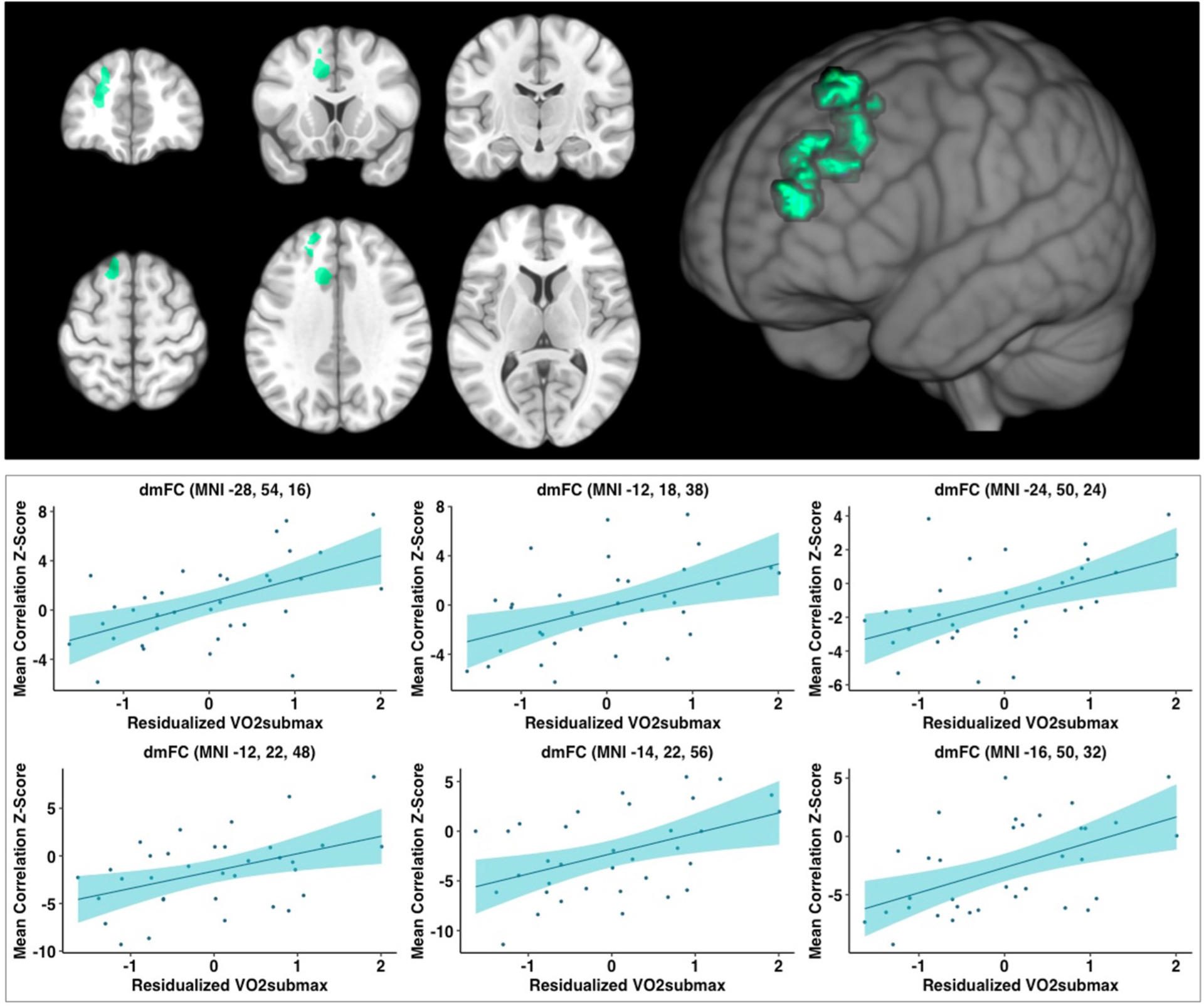
Analyses revealed a large significant cluster of greater connectivity to the hippocampus, as a function of higher cardiorespiratory fitness level, overlapping with regions in the dmFC. Analyses covaried for age, education, and framewise displacement. Peak values (z-max) were 4.37, 3.86, 3.51,3.44, 3.39, and 3.33; all ps < 0.05, respectively. Scatterplots depict the relationship between cardiorespiratory fitness and hippocampus-to-ROI signal covariation at rest. Mean framewise displacement, age, and education were regressed out of cardiorespiratory fitness values prior to plotting. dmFC, dorsomedial frontal cortex; MNI, Montreal Neurological Institute (coordinates).

When controlling for age, education, and framewise displacement, higher cardiorespiratory fitness (VO_2_submax represented in ml/kg/min) was associated with greater rsFC of the hippocampus to a single cluster encompassing the left dorsomedial frontal cortex (dmFC), including the anterior cingulate cortex (ACC), supplementary motor area, medial frontal gyrus, as well as dorsolateral portions of the PFC including superior frontal gyrus and middle frontal gyrus (voxel-wise z-max = 4.37, *p* =0.003, cluster extent = 1,020 voxels). For illustration purposes, we created six ROI spheres surrounding the peak voxels of the cluster (*z*-max_1_ = 4.37, z-max_2_ = 3.86, *z*-max_3_ = 3.51, *z*-max_4_ = 3.44; *z*-max_5_ = 3.39, *z*-max_6_ = 3.33; all ps < 0.05; Table 3). Higher levels of cardiorespiratory fitness were associated with increased signal between the hippocampus and each of these ROIs, as illustrated in Figure 1. Peak voxel spheres were used as ROIs in the subsequent analyses.

Sensitivity analyses excluding the three participants who started AI therapy more than 3 weeks prior to completing baseline MRI generated results that were consistent with primary analyses. Specifically, when controlling for age, education, and framewise displacement, higher cardiorespiratory fitness was associated with greater rsFC of the hippocampus in a single cluster (voxel-wise z-max = 4.15, *p* = 0.006, cluster extent = 884 voxels) with six peak ROI spheres (*z*-max_1_ = 4.15, *z*-max_2_ = 3.85, *z*-max_3_ = 3.42, *z*-max_4_ = 3.30; *z*-max_5_ = 3.28, *z*-max_6_ = 3.16; all ps < 0.05).

### Cardiorespiratory fitness and cognitive performance

Cardiorespiratory fitness was not significantly associated with any of the composite cognitive factors: learning and Memory (*B* = −0.01, *p* = 0.83), Verbal Memory (*B* = −0.01, *p* = 0.81), Attention (*B* = −0.02, *p* = 0.66), Executive Function (*B* = 0.04, *p* = 0.34), or Mental Flexibility (*B* = 0.02, *p* = 0.56), Psychomotor Speed (*B* = −0.04, *p* = 0.42), or Working Memory (*B* = −0.06, *p* = 0.15).

Sensitivity analyses excluding the three participants who started AI therapy prior to completing baseline cognitive testing generated results that were consistent with primary analyses. Cardiorespiratory fitness was not significantly associated with any of the cognitive composite factors.

### Seed-to-ROI rsFC and cognitive performance

Resting state connectivity values between the hippocampal seed and each of the six ROIs in the dmFC were not significantly associated with any of the composite cognitive factors: Learning and Memory (*B* range = −0.06–0.05, *p*-value range = 0.12–0.89), Verbal Memory (*B* range = −0.06–0.07, *p*-value range = 0.06–0.80), Attention (*B* range = −0.06–0.04, *p*-value range = 0.25–0.92), Executive Function (*B* range = −0.02–0.04, *p*-value range = 0.14–0.95), Mental Flexibility (*B* range = −0.03–0.002, *p*-value range = 0.34–0.99), Psychomotor Speed (*B* range = −0.02–0.04, *p*-value range = 0.15–0.85), or Working Memory (*B* range = −0.05–0.002, *p*-value range = 0.14–0.95).

Sensitivity analyses excluding the three participants who started AI therapy prior to completing both the baseline cognitive testing and MRI assessments generated results that were consistent with primary analyses (i.e., nonsignificant). However, there were marginally significant associations between the resting state connectivity values for the ROI located within the premotor and supplementary motor area (*B* = 0.08, *p* = 0.052) and the ROI located within the dorsolateral PFC (*B* = −0.08, *p* = 0.051) and verbal memory performance.

## Discussion

To our knowledge, this study is the first to examine associations between cardiorespiratory fitness, rsFC, and cognitive performance in postmenopausal women with breast cancer. Consistent with our predictions, higher levels of cardiorespiratory fitness were associated with heightened connectivity between the hippocampus and medial frontal regions of the brain, most prominently the anterior cingulate cortex (ACC), supplementary motor area, medial frontal gyrus, as well as dorsolateral portions of the PFC including superior frontal gyrus and middle frontal gyrus. However, contrary to our expectations, connectivity in these regions was not correlated with cognitive performance.

Prior research has indicated that, compared to matched controls, women with breast cancer have poorer cognitive function at pre-treatment (Ahles et al., 2008; Bender et al., 2015). Further, recent work suggests that heterogeneity in structural and functional brain reserve may predict susceptibility to further decline following breast cancer treatment. For instance, one study revealed that lower white matter microstructural integrity at baseline predicted cognitive decline following chemotherapy (de Ruiter et al., 2021). Thus, characterizing baseline brain health in women with breast cancer may serve an important role in predicting their resilience against cancer-related cognitive impairment; furthermore, identifying modifiable factors associated with greater neural integrity at baseline may be central in early prevention efforts targeting cancer-related cognitive impairment. In the current study, we identified cardiorespiratory fitness as significantly associated with connectivity between the hippocampus and multiple frontal brain regions, suggesting that women with greater cardiorespiratory fitness at baseline may also benefit from higher functional brain reserve prior to undergoing adjuvent therapy.

In particular, the medial frontal lobe subserves a number of critical higher-level cognitive functions including executive control and attention allocation (Hwang et al., 2015; Feng et al., 2018). Moreover, the medial prefrontal cortex communicates with the hippocampus to support learning and memory retrieval (Euston et al., 2012). These cognitive faculties have been shown to be susceptible to decline in numerous neurocognitive disorders of the aging brain (Jobson et al., 2021) and following breast cancer treatment (Bergouignan et al., 2011; de Ruiter et al., 2011; Kesler et al., 2013), underscoring the importance in promoting the functional health of these regions prior to breast cancer treatment.

Furthermore, there have been numerous rsFC studies which have found weakened connectivity between the hippocampus and medial PFC in populations with prodromal or preclinical Alzheimer’s Disease, APOE4 carriers, mild cognitive impairment (MCI), and mild dementia (Jobson et al., 2021). These studies suggest that heightened connectivity between the hippocampus and dmFC is cognitively adaptive. However, some studies find that certain patterns of heightened hippocampal connectivity may be maladaptive. For example, Gardini et al. found that, in individuals with MCI, increased connectivity between these regions was associated with worse semantic memory performance (Gardini et al., 2015). Similarly in a sample with breast cancer, Cheng et al. (2017) found that patients (*N* = 34; M_age_ = 52 ± 8.48) who underwent chemotherapy exhibited higher hippocampal connectivity, relative to controls, to the frontal and parietal cortex, posterior cingulate cortex, precuneus, middle occipital gyrus, and the cerebellum, which in turn correlated with poorer cognitive performance (Cheng et al., 2017). In the present study, we speculate that greater connectivity between the hippocampus and dmFC is beneficial for brain health, since this pattern was associated with increased cardiorespiratory fitness which is consistently linked to better brain health and function in both healthy and clinical populations. However, since these cardiorespiratory fitness-related connectivity patterns were not significantly associated with the cognitive performance measures, it is unclear to what extent they represent meaningful brain health diffrences, pointing toward an avenue for future research.

Despite significant associations between cardiorespiratory fitness and hippocampal-frontal rsFC, these cardiorespiratory fitness-related connectivity patterns were not significantly associated with the cognitive performance measures. One possibility accounting for this pattern of findings is that measuring rsFC may provide a more sensitive measurement of early differences in brain health, which may make a difference in both aging-related cognitive decline and in breast cancer progression and treatment. Our sample of 34 participants may have also been too small to detect any association with cognitive performance. In fact, the larger study sample from which this neuroimaging study recruited from did reveal associations between cardiorespiratory fitness and cognitive performance in the domains of processing speed and verbal memory (Bender et al., 2020), consistent with prior studies conducted in healthy older adults (Aghjayan et al., 2021), suggesting that the present substudy might have been underpowered to reveal associations with the cognitive data. Still, it should be noted that our analyses of the relationship between extracted hippocampal-frontal connectivity values and cognitive performance yielded relatively small standardized regression coefficients; therefore, these associations may not be meaningfully influenced by an increased sample size. Nevertheless, our findings of an association between cardiorespiratory fitness and hippocampal rsFC highlight the importance of longitudinal follow-up analyses exploring whether hippocampal rsFC at baseline may predict change or resilience to cancer-associated cognitive impairment over time.

Overall, our findings suggest that having higher cardiorespiratory fitness may enhance the functional organization of the hippocampus, which is thought to exhibit maladaptive patterns of connectivity in breast cancer. However, our study had several important limitations. As noted above, our sample size was small, and our study was cross-sectional. As such, we can only speculate about the causal relationship between cardiorespiratory fitness and rsFC. These participants went on to be randomly assigned to either a 6-month exercise intervention or control group, followed by another MRI, so it will be important to analyze those data to determine whether exercise-related increases in cardiorespiratory fitness were associated with increases in rsFC between the hippocampus and dmFC. If so, then we can be more confident in our interpretation of our results, which suggest that higher cardiorespiratory fitness may confer neural benefits for postmenopausal women with breast cancer. Despite the numerous cognitive correlates of the hippocampus and medial frontal lobe, we are also notably limited in our ability to speculate about the cognitive implications of our rsFC findings given that associations between hippocampal-frontal connectivity and cognitive performance were nonsignificant in this sample. In addition, as we focused entirely on objective measures of cognitive performance in this study, incorporating measures of self-reported cognitive function may be an important avenue for future analyses, particularly as measures of subjective cognitive decline may reveal early changes in cognition that are not yet evident on objective testing.

It is also important to note that the present study did not include a cancer-free control group. Thus, we are limited in our ability to conclusively state whether our findings are truly specific to women with breast cancer. The participants in this sample were older, so it is possible our pattern of results may reflect the effects of aging rather than breast cancer, specifically. Yet, some literature suggests that patients with breast cancer may experience an accelerated aging trajectory (Bender et al., 2018), therefore the effects of aging and cancer on the brain may not be mutually exclusive. Further, an extensive literature suggests that high body mass index (BMI) is associated with greater risk of poor outcomes in breast cancer and increased cognitive decline (Protani et al., 2010; Neuhouser et al., 2015; Dye et al., 2017). As our cardiorespiratory fitness measure accounts for body weight in kilograms, we did not adjust for BMI in primary analyses, although additional sensitivity analyses including BMI as a covariate generated a similar pattern of findings with respect to hippocampal-frontal rsFC and cognitive outcomes (results not shown). An additional limitation is that there were participants who had begun their AI therapy prior to undergoing the MRI. It is possible that their treatment altered their rsFC; however, in sensitivity analyses excluding these participants our results did not significantly change. Finally, fMRI and thus our measurement of rsFC is itself a correlative measure; it measures blood oxygenation, which is thought to represent underlying neuronal activity, but it does not directly measure the neuroelectrical activity of neurons. However, despite these limitations, our study fills an important gap in the literature as so little is known about the relationship between cardiorespiratory fitness and rsFC of the hippocampus in women with breast cancer.

## Conclusion

In sum, this study provides the first evidence, to our knowledge, that cardiorespiratory fitness is associated with rsFC of the hippocampus in postmenopausal women with breast cancer. We demonstrated that higher cardiorespiratory fitness was associated with greater rsFC of the hippocampus to six clusters within the dorsomedial frontal cortex, a connectivity pattern which in other populations has been shown to be cognitively beneficial. Although these connectivity patterns were not significantly associated with cognitive performance in our sample, they may predict long-term cognitive resilience. Future longitudinal studies are needed to determine whether higher cardiorespiratory fitness is cognitively protective over the course of breast cancer treatment, and whether increasing cardiorespiratory fitness via participation in exercise can increase rsFC in this population. Cardiorespiratory fitness is consistently linked with beneficial neurocognitive outcomes in numerous populations; our study indicates that it may be beneficial in women with breast cancer as well, potentially reducing the vulnerability of the brain to the deleterious effects of breast cancer and its treatment.

## Figures and Tables

**TABLE 1:** Exploratory factor analysis was used to reduce neurocognitive measures to seven composite factors.

1. Learning and memory
Auditory Verbal Learning Test (short-delay correct, long-delay correct, total correct) CANTAB Paired Associate Learning (1st attempt memory score, total errors adjusted) Rey Complex Figure (immediate and delayed recall)
2. Verbal memory
Rivermead Story (immediate and delayed recall)
3. Attention
CANTAB Rapid Visual Information Processing (A’, mean response latency) DKEFS Color Word Interference Test (Condition 3—inhibition raw score, Condition 4—inhibition switching raw score); Digit Symbol Substitution Test (raw score); Verbal Fluency Test (total correct)
4. Mental flexibility
Trail Making Test: Part B (time, errors)
5. Executive function
CANTAB One Touch Stockings of Cambridge (mean choices to correct moves, problems solved on first choice)
6. Working memory
CANTAB Spatial Working Memory (between errors [trials 4, 6, and 8] and strategy)
7. Psychomotor speed
Grooved Pegboard (dominant and non-dominant hand time to insert) Digit Vigilance Test (total time and errors)

CANTAB, Cambridge Neuropsychological Test Automated Battery; DKEFS, Delis-Kaplan Executive Function System.

**TABLE 2:** Demographic, clinical, and cognitive characteristics of the sample (*N* = 34).

Demographics	
Years of age [*M* ± *SD*]	63.59 ± 5.73
Years of education [*M* ± *SD*]	15.94 ± 2.88
VO2submax (ml/kg/min) [*M* ± *SD*]	16.78 ± 2.70
Body Mass Index (kg/m^2^)	31.83 ± 6.62
**Race** *[N* **(%)]**	
White	29 (85)
Black or African American	5 (12)
American Indian	2 (6)
Multi-racial	2 (6)
Hispanic ethnicity	1 (3)
**Clinical features** *[N* **(%)]**	
Overall cancer stage	
Stage 0 DCIS	10 (29)
Stage 1	17 (50)
Stage 2a	7 (21)
**T-stage [*N* (%)]**	
Tis	10 (29)
T1a	2 (6)
T1b	8 (24)
T1d	9 (27)
T2	5 (15)
Started adjuvant therapy within 3 weeks of MRI* [N (%)]	31 (91)
**Cognitive composite Z-scores [*M* ±*SD*]**	
Learning and memory	−0.04 ± 0.81
Verbal memory	−0.30 ± 0.95
Attention	−0.35 ± 0.84
Executive function	0.20 ± 0.61
Mental flexibility	0.20 ± 0.47
Psychomotor speed	0.10 ± 0.82
Working memory	−0.41 ± 0.64

* The three participants who underwent baseline MRI at least 3 weeks after starting adjuvant treatment completed imaging 45, 219, and 525 days following the start of treatment, respectively.

**TABLE 3:** MNI coordinates (mm) of local maxima in regions of interest for which CRF was positively associated with resting state functional connectivity with the hippocampal seed.

Region of Interest	Hemisphere	Local maxima z-score	*x*	*y*	*z*
Anterior PFC	Left	4.37	−28	54	16
Anterior cingulate cortex	Left	3.86	−12	18	38
Anterior PFC	Left	3.51	−24	50	24
Medial frontal gyrus	Left	3.44	−12	22	48
Premotor + supplementary motor area	Left	3.39	−14	22	56
Dorsolateral PFC	Left	3.33	−16	50	32

Total cluster extent = 1,020 voxels (*p* = 0.003).

Regions met a cluster-defining threshold of *z* > 2.3 and *p* < 0.05.

MNI, Montreal Neurological Institute; CRF, cardiorespiratory fitness; PFC, prefrontal cortex.

## Data Availability

The raw data supporting the conclusions of this article will be made available by the authors, without undue reservation.
